# Two-Dimensional SERS Sensor Array for Identifying and Visualizing the Gas Spatial Distributions of Two Distinct Odor Sources

**DOI:** 10.3390/s24030790

**Published:** 2024-01-25

**Authors:** Lin Chen, Hao Guo, Cong Wang, Bin Chen, Fumihiro Sassa, Kenshi Hayashi

**Affiliations:** 1 Department of Information Science, Joint Graduate School of Mathematics for Innovation, Kyushu University, Fukuoka 819-0395, Japan; 2 Department of Electronics, Graduate School of Information Science and Electrical Engineering, Kyushu University, Fukuoka 819-0395, Japan; guo.hao.097@s.kyushu-u.ac.jp (H.G.); cong.929@s.kyushu-u.ac.jp (C.W.); sassa@ed.kyushu-u.ac.jp (F.S.); 3 Chongqing Key Laboratory of Non-Linear Circuit and Intelligent Information Processing, College of Electronic and Information Engineering, Southwest University, Chongqing 400715, China; chenbin121@swu.edu.cn

**Keywords:** two-dimensional surface-enhanced Raman scattering sensor array, non-negative matrix factorization, gas spatial distribution, localization of the odor source, size estimation, visualization of two distinct odor sources

## Abstract

The spatial distribution of gas emitted from an odor source provides valuable information regarding the composition, size, and localization of the odor source. Surface-enhanced Raman scattering (SERS) gas sensors exhibit ultra-high sensitivity, molecular specificity, rapid response, and large-area detection. In this paper, a SERS gas sensor array was developed for visualizing the spatial distribution of gas evaporated from benzaldehyde and 4-ethylbenzaldehyde odor sources. The SERS spectra of the gas were collected by scanning the sensor array using an automatic detection system. The non-negative matrix factorization algorithm was employed to extract feature and concentration information at each spot on the sensor array. A heatmap image was generated for visualizing the gas spatial distribution using concentration information. Gaussian fitting was applied to process the image for localizing the odor source. The size of the odor source was estimated using the processed image. Moreover, the spectra of benzaldehyde, 4-ethylbenzaldehyde, and their gas mixture were simultaneously detected using one SERS sensor array. The feature information was recognized using a convolutional neural network with an accuracy of 98.21%. As a result, the benzaldehyde and 4-ethylbenzaldehyde odor sources were identified and visualized. Our research findings have various potential applications, including odor source localization, environmental monitoring, and healthcare.

## 1. Introduction

The detection and visualization of volatile organic compounds (VOCs) have significant applications in various fields [1], including environmental monitoring [2], biotechnology [3], food safety [4,5], adulteration detection [6,7], and healthcare [8,9]. Furthermore, the spatial distribution of the VOCs’ gases can provide significant information about the odor source. By analyzing the spatial distribution of the detected gas influenced by airflow, we can use a robot equipped with recognition algorithms to determine the location of the odor source [10,11,12]. Additionally, through the analysis of the spatial distribution of gas evaporating from the odor source, we can clarify the information contained in the odor source, including composition, localization, and temporal variations [13,14,15,16].

The spatial distribution of gas can be visualized by utilizing the gas concentration information obtained from various positions across the sensors. Metal oxide semiconductor (MOS) sensors, utilizing materials like SnO_2_, CuO, WO_3_, and ZnO, have found extensive use for gas detection [17]. These sensors display alterations in the resistance corresponding to fluctuations in the gas concentration. Li et al. utilized CuO nanowires for fabricating an MOS sensor through template-assisted electrodeposition, achieving a detection limit of 2.5 parts per billion (ppb) for H_2_S gas [18]. Despite their high sensitivity and rapid response times, MOS sensors encounter challenges related to high operational temperature requirements. Quartz crystal microbalance (QCM) sensors measure gas mass and are utilized for gas concentration determinations. Due to their lack of selectivity, these sensors benefit from coatings of selective materials, like polymers [19], carbon nanotubes [20], or molecularly imprinted polymers (MIPs) [21]. Yang et al. used a porous MIP film on a QCM sensor to detect formaldehyde gas, showcasing its selectivity in the presence of hydrogen chloride and hydrogen fluoride [21]. QCM sensors boast high sensitivity, quick response times, and room temperature operation capabilities. However, their detection accuracy can be impacted by humidity. Fluorescent sensors, utilizing fluorescent materials, are optical sensors employed for gas detection [22]. Interactions between target gases and fluorescent materials induce changes in the fluorescence properties, such as intensity, emission, or decay, enabling the measurement of the gas concentration [1]. Petruci et al. introduced a portable online sensor platform employing fluorescein mercury acetate for gaseous hydrogen sulfide detection purposes, demonstrating a linear calibration within the range of 17–67 ppb and a 3 ppb detection limit [23]. While fluorescent sensors are widely acknowledged for their sensitivity and specificity, they require tailored materials for target gases, which can pose toxicity concerns. Surface-enhanced Raman scattering (SERS) has emerged as an efficient gas sensing technique, enhancing the Raman signals of molecules adsorbed onto metal surfaces, like silver or gold nanoparticles (NPs) [24,25]. The Raman signal contains unique molecular fingerprints, enabling selective gas detection [26]. Additionally, SERS sensors possess high sensitivity, even at the single-molecule detection level [27], allowing for the detection of low-concentration gases. Furthermore, immediately after the laser irradiation of the gas-adhered SERS sensor, the SERS spectra of the gas can be promptly collected, showcasing the rapid response characteristics of the SERS sensor [28].

In this work, we develop a two-dimensional (2D) SERS sensor array to visualize the spatial distribution of gas evaporating from odor sources placed at different positions. Benzaldehyde (BZD) and 4-ethylbenzaldehyde (EBZD) are chosen as the odor sources. The SERS spectra of the gas adsorbed on the sensor are acquired by scanning the sensor array and decomposed using the non-negative matrix factorization (NMF) algorithm to extract the feature and concentration information of the detected gas. The feature information corresponds to the SERS spectra of the gas. The concentration information is used to visualize the gas spatial distribution by creating a heatmap image. Then, the Gaussian fitting method is employed to process the heatmap image for localizing the odor source. The odor sources of the same analyte placed at different positions are accurately localized. The visualization result is also used to estimate the size of the odor source. The estimated and actual sizes show a strong linear correlation, with a correlation coefficient (R^2^) of 0.968. Additionally, BZD and EBZD odor sources are simultaneously identified and localized using one SERS sensor array. The SERS spectra of these two gases are correctly identified using a convolutional neural network (CNN) model, with a classification accuracy of 98.21%. This gas sensing method demonstrates significant potential for visualizing the spatial distribution of VOC gases, which can be used to localize an odor source, monitor environmental pollution, and provide good healthcare.

## 2. Related Work

To visualize the spatial distribution of gases, the sensor technology employed should meet three primary conditions. Firstly, it should be an array-type sensor: arranging multiple sensors in an array allows for gas detection at various positions, aiding in capturing the comprehensive spatial distribution information of the gas. Secondly, the sensor requires high sensitivity: gas concentrations are typically low and exhibit considerable mobility, necessitating highly sensitive sensors to swiftly detect their presence. Thirdly, the sensor ought to possess an exceptional spatial resolution: capturing subtle variations in the gas distribution and enhancing the comprehensibility of visualization results requires sensor technology with a high spatial resolution.

A large-scale sensor array had been previously assembled using QCM sensors, serving to visualize gas distributions. Ishida et al. developed an olfactory video camera comprising a sensor array utilizing 21 QCM sensors to track the flow of triethylamine gas [29]. Each sensor in this array measured 4 mm × 8mm, with a spacing of 12.7 mm between the sensors. Consequently, the entire sensor array occupied an area of approximately 90.8 mm × 70.8 mm. This portable and cost-effective sensing system was capable of visualizing gas flow speeds of up to 30 cm/s. However, due to the limited amount of data collected, the resulting images had a relatively low resolution.

Iitani et al. developed a fluorometric sensor for visualizing the gas distribution [13]. They designed a 90 mm × 90 mm alcohol dehydrogenase (ADH)-immobilized mesh to detect transcutaneous ethanol gas concentrations. When gaseous ethanol encountered the mesh soaked with an oxidized nicotinamide adenine dinucleotide (NAD) solution, an ADH-mediated reaction produced a reduced form of NAD (NADH). NADH emitted fluorescence under ultraviolet excitation. Consequently, the distribution of gaseous ethanol concentrations was visualized by capturing the fluorescence intensity distribution using a camera. A high-resolution color image visualizing the spatial distribution of the detected gas was obtained. A fluorometric sensor necessitates suitable fluorescent dyes for the target gas and requires a consideration of environmental lighting interference.

Matsuoka et al. utilized a gas sensor employing localized surface plasmon resonance (LSPR) in conjunction with a cooled charge-coupled device camera to visualize gas distributions [30]. The LSPR sensor was easily manufactured by depositing metallic nanoparticles (NPs) on a 50 mm × 50 mm substrate, requiring no further modification, demonstrating a rapid response. Specific gas molecules interacting with the NPs induced a shift in the LSPR frequency, enabling the detection and quantification of gases. The change in LSPR frequency was used for gas visualization. In their results, four gases, including geraniol, eugenol, piperitone, and pentadecane, were identified and visualized using an LSPR sensor. However, the selectivity of the LSPR sensor remains unresolved.

Notably, SERS sensors can be used to address several challenges associated with other gas sensing technologies. SERS sensors have three main advantages: (1) the correct identification of the analytes by vibrational spectroscopy [26,31], (2) label-free molecular detection, which simplifies the detection process [24], and (3) high-resolution imaging by capturing SERS spectra at different positions on the sensor surface [32,33].

## 3. Materials and Methods

In this section, we delineated the method for fabricating our SERS sensor and evaluating its reproducibility performance. Additionally, we provided details for detecting and visualizing the spatial distribution of gases evaporating from the odor source.

### 3.1. Fabrication of SERS Sensor

The SERS sensor was fabricated using silver nanoparticles (Ag NPs), which were synthesized according to a previous report [34]. First, the Ag NP seeds solution was synthesized, as described in the Supporting Information. Second, Ag NPs with a size of approximately 90 nm were synthesized using the prepared Ag seeds solution. Then, a densely packed Ag NPs monolayer film was formed using an oil/water/oil three-phase system based on the Marangoni effect [35,36]. Finally, a glass substrate was inserted under the monolayer film at an angle and then pulled out to transfer the film onto the glass substrate (5 mm × 5 mm). The whole fabrication process is shown in Figure S1. Nine SERS sensors were arranged in a 3 × 3 array to construct a 2D sensor array. Within the sensor array, adjacent sensors were positioned in close proximity, with their distance was considered negligible (0 mm). As a result, the overall size of our SERS sensor array measured 15 mm × 15 mm.

### 3.2. Reproducibility of the Fabricated Sensor

Nine SERS sensors were immersed in 8 mL of a 4-aminothiophenol (4-ATP) ethanol solution (1 µM) for 1 h. After that, the sensors were cleaned with the ethanol solution and dried under a flow of nitrogen. The SERS intensities of the selected characteristic peak were calculated to evaluate the reproducibility of the fabricated SERS sensor.

### 3.3. Detection of the Gas Evaporating from Odor Sources

We individually added BZD or EBZD solutions to an aluminum cup as odor sources. The mixed odor source was prepared by mixing EBZD and BZD solutions with a volume ratio of 1:1.

The diameter and depth of the cup were both 5 mm. A Peltier device was used to heat the odor source, thereby accelerating the evaporation of the gas. Herein, the BZD, EBZD and EBZD/BZD solutions were heated for one, two, and two minutes, respectively. Five positions were designated for placing the odor source, namely the center, left-bottom, left-up_left-bottom, right-up_left-bottom and center_left-bottom, as shown in Figure S2. The constructed SERS sensor array was placed above the stationary odor source, which was fixed in a selected position in an enclosed space. The detection process is illustrated in Figure S3. After heating, the sensor array was moved from the odor source and fixed in the detection chamber. The SERS spectra of the gas were collected by scanning the sensor array using a program-controlled detection system (Figure S4). In the detection process, 1296 spectra from one sensor array were obtained in a scanning format of 36 points × 36 points.

The odor sources with different sizes were detected using the same method. An aluminum plate with one circular hole was placed above the odor source, and the diameter of the hole was changed to alter the size of the odor source. In addition, the centers of the hole and the odor source were aligned along the same vertical line, as shown in Figure S5.

### 3.4. Visualization of the Gas Spatial Distribution

On the surface of the sensor array, areas closer to the odor source exhibited greater gas adsorption, whereas those farther away had relatively less gas adsorption. Thus, the spatial distribution of gases was visualized by considering the amount of gas adsorption on the sensor surface. NMF was used to decompose a non-negative matrix into the product of a feature matrix and a weight matrix [37,38].

In this study, the V matrix was constructed by applying the SERS spectra obtained from n points on the SERS sensor array. The dimension of each spectrum was m. Therefore, V was decomposed as:

(1) $$V_{n\times m}=W_{n\times r}H_{r\times m}$$

where matrix, W, represents the concentration of each component at each position, matrix H represents the feature of each component, and r is the number of the components extracted from the sensor array. For component 1, $h_{1\times m}$ is the feature of the component and $w_{n\times 1}$ is the concentration of component 1 in each position. Therefore, $w_{n\times 1}$ is reconstructed as a matrix for visualizing the gas spatial distribution, generating a heatmap image. Finally, the component was identified by the $h_{1\times m}$ feature result.

In the experiment, several factors affected the measurement, including the air interference and randomness of the gas flow. The concentration information was not considered ideal. Thus, the Gaussian fitting model was used to process this image (see Supporting Information) [39].

### 3.5. Construction of the Datasets for the CNN Model

The SERS spectra matrix obtained from detecting each odor source was utilized to establish the datasets. For the training dataset, the SERS spectra matrix results were obtained by placing the BZD odor source in the center, left-top, and left-bottom positions, and similarly for the EBZD source. Moreover, we obtained spectra matrix results by detecting two BZD odor sources positioned in three different patterns using a single sensor array. The obtained SERS spectra matrices underwent decompositions through the non-negative matrix factorization algorithm, resulting in a three-feature information matrix that included features for the target gas, baseline, and noise, respectively. Within the feature matrix, the feature information values linked to BZD and EBZD gases were labeled as 1 and 2, respectively, while the other information attributed to the noise and baseline (interference) was labeled as 0. Therefore, this resulted in 21 samples for the BZD gas, 13 for the EBZD gas, and 68 for interference in the training datasets. Each feature information (spectra) consisted of 307 Raman shifts. Regarding the testing dataset, the SERS spectra matrices collected from BZD and EBZD detections using a single SERS sensor array were composed to extract four features for the target gases (two) and interference (two). We acquired 14 SERS spectra matrix results by placing these two odor sources in three different positional patterns. Thus, the testing dataset comprised 14 samples each for BZD and EBZD, and 28 samples for the interference.

### 3.6. Identification of the Odor Source Using a CNN Model

A CNN model was used to identify the component in the NMF decomposition result. We utilized Python (version 3.8) and PyTorch (version 2.1) to construct our CNN model. The training datasets were constructed from the feature data of the component when one and two of the same odor sources were detected. The training dataset was divided into training and validation sets at a ratio of 7:3. The input data were fed into a one-dimensional convolutional layer, followed by a rectified linear units (ReLUs) layer. Two consecutive fully connected layers were attached to a ReLU layer. Finally, the output was classified by the SoftMax layer. Therefore, there were five layers in the CNN model. At the beginning of the training process, the learning rate was 0.0001 and the adaptive moment estimation optimizer was selected. During the training phase, a cosine annealing learning rate scheduling strategy was employed with a period of 10 epochs and a minimum learning rate of 0.00001. The trained CNN model was employed to identify the components in the NMF decomposition result upon the detection of two distinct odor sources.

## 4. Results and Discussion

In this section, we presented the morphology and performance results of the fabricated SERS sensor. We compared the SERS spectra of target gases detected by the SERS sensor array. Additionally, we employed the non-negative matrix factorization algorithm to decompose the SERS spectra matrix for identifying and visualizing the spatial distribution of gas evaporation from the odor source.

### 4.1. Performance of Fabricated SERS Sensor

**Figure 1:**
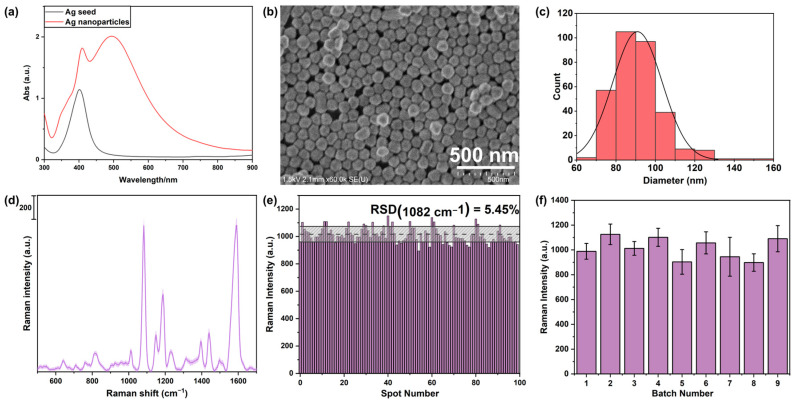
(**a**) The UV-vis spectra of the synthesized Ag nanoparticle (NP) seeds and Ag NPs with large sizes. (**b**) Scanning electron microscope (SEM) image of the Ag NPs on the SERS sensor. (**c**) Size distribution of the Ag NPs obtained by ImageJ software. (**d**) SERS spectra of 4-aminothiophenol (4-ATP) modified on the fabricated SERS sensor. (**e**) The histogram of the SERS intensities at 1082 cm^−1^ in their corresponding SERS spectra of 4-ATP collected from 100 positions on the sensor. (**f**) Average values of SERS intensities at 1082 cm^−1^ of 4-ATP obtained from nine SERS sensors.

SERS sensors were fabricated by transferring the Ag NP monolayer film to the glass substrate using an oil/water/oil three-phase system. The Ag NPs with large sizes were synthesized using the seed-mediated growth method. Ultraviolet-visible (UV-vis) spectra were obtained to understand the optical properties of the Ag NP seeds and Ag NPs, as shown in Figure 1a. A distinct dipole peak was observed at approximately 400 nm for the Ag seeds. As for the Ag NPs, a new quadrupole peak was observed at around 500 nm. Furthermore, the dipole peak position was red-shifted to a longer wavelength [40]. The morphological features of the Ag NPs on the SERS sensor are shown in Figure 1b, demonstrating that the monolayer film with a dense and large-scale arrangement is transferred to the glass substrate. The sizes of the synthesized Ag NPs were almost identical. The size distribution of the Ag NPs on the sensor was obtained by processing the scanning electron micrograph (SEM) image using ImageJ software (version 1.53), as illustrated in Figure 1c. The average diameter of the Ag NPs was estimated to be 90.90 ± 12.56 nm.

To evaluate the uniformity and reproducibility of the fabricated SERS sensor, the sensor was immersed in a 4-ATP solution, and the SERS spectrum of 4-ATP was recorded, as shown in Figure 1d. The bond vibration information of the prominent characteristic peaks is summarized in Table S1 [28]. We collected 100 SERS spectra with a step distance of 200 μm over an area of 2 mm × 2 mm. The spot-to-spot variation distributions of the SERS intensities for the 1082 cm^−1^ peak are shown in Figure 1e. The relative standard deviation (RSD) for the SERS intensity was 5.45%. Therefore, the fabricated SERS sensor presented high uniformity over a large area [31,41,42]. Additionally, the reproducibility of the SERS sensor was investigated using nine batches of sensors modified by 4-ATP. The average SERS intensities of 100 spectra at 1082 cm^−1^ from nine batches of fabricated sensors are summarized in Figure 1f. The RSD value for nine sensors was 8.47%, which confirmed the high batch reproducibility of the SERS sensor. Stability is a crucial parameter for assessing the performance of SERS sensors. The fabricated SERS sensor can be used after being stored in a vacuum box for one week. In this study, our primary focus was on the reproducibility of the sensors, a characteristic significantly affecting the spatial visualization of gases. We used a new SERS sensor for each visualization experiment, and due to the difficulty of removing adsorbed gases, we did not recycle or reuse the substrates.

### 4.2. SERS Spectra of the Gas Adsorbed on the 2D Sensor Array

**Figure 2:**
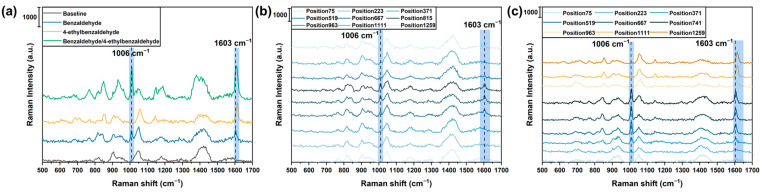
(**a**) The SERS spectra of the sensor baseline, benzaldehyde (BZD), 4-ethylbenzaldehyde (EBZD), and mixture of benzaldehyde and 4-ethylbenzaldehyde gases. (**b**) The SERS spectra were collected from the spot on the diagonal line of the SERS sensor array when the BZD odor source placed at the center position was detected, and (**c**) the BZD odor source placed at the center position and EBZD odor source placed at the left-bottom position were simultaneously detected.

After the 2D SERS sensor array was placed above and facing the heated BZD and EBZD odor sources for 1 or 2 min, the gas-adsorbed sensor array was positioned inside a detection chamber and scanned to collect SERS spectra. The SERS spectra of the SERS sensor baseline and three types of gases are shown in Figure 2a. Two characteristic peaks were observed at 1006 and 1603 cm^−1^ for the BZD gas [43]. For the EBZD gas, only one distinct peak appeared at 1614 cm^−1^ [44]. Notably, the SERS spectra of the gas mixture containing these two gases exhibited peaks at 1010 and 1607 cm^−1^. The peak at 1010 cm^−1^ was attributed to the BZD gas. The spectrum resolution of our Raman spectrometer was approximately 4 cm^−1^. The characteristic peaks observed at 1603 cm^−1^ and 1614 cm^−1^ corresponded to the BZD gas. Within the range from 1603 cm^−1^ to 1614 cm^−1^, only two values were present, resulting in the detection of a single peak (1607 cm^−1^) within this range. Ultimately, the distinct spectra can be used to distinguish the different gases.

In the detection procedure, 1296 SERS spectra were obtained by scanning the 2D sensor array in a format of 36 spots × 36 spots. The sensor array was scanned from the top-left corner to the bottom-right corner. By comparing the spectra along the diagonal of the sensor array, the variations in the SERS spectra with respect to the sensor’s position were confirmed (Figure 2b). In this result, the BZD odor source was positioned in the central location. The SERS intensities of the spectra obtained from the central location were stronger than those from the surrounding locations. Therefore, the variation in SERS spectra among different locations could be used to visualize the spatial distribution of the gas.

### 4.3. Visualization of the Spatial Distribution of the Gas Evaporating from the Odor Source

A total of 1296 SERS spectra were collected for each detection, and each spectrum consisted of 308 different Raman shift features, resulting in the detection result matrix of $V_{1296\times 308}$. As shown in Figure 2b, even when the patterns of the SERS spectra were identical, the SERS intensities were affected by the gas concentrations. Hence, the matrix, V, was decomposed into a concentration matrix, W, and a feature matrix, H, using the NMF algorithm [45]. As described in Section 3.4, the tunable parameter, r, should be optimized to accurately extract the concentration information contained in matrix, V. For a single odor source, there were three types of spectra in matrix V, namely those for the sensor baseline, gas, and noise. Owing to the slight gap between the sensors in the sensor array setup, some spectra were collected from the space between the two sensors. These spectra were considered as noise in the matrix, V. Therefore, the parameter, r, was set to 3 in the NMF algorithm for the detection of a single odor source. In this study, the value of r was determined by adding 2 to the number of gas types.

**Figure 3:**
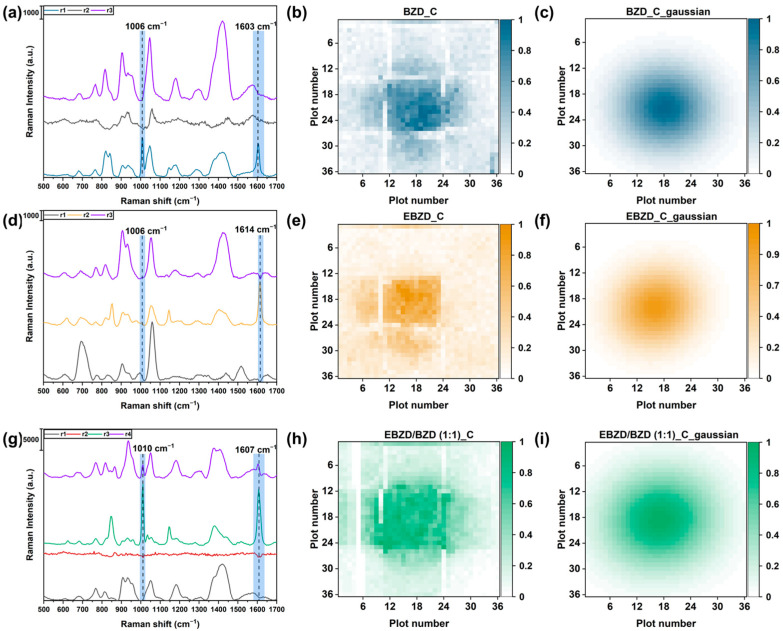
(**a**) The spectra of in the feature matrix, H, after the detection result matrix, V, was decomposed using the non-negative matrix factorization (NMF) algorithm for (**a**) benzaldehyde (BZD), (**d**) 4-ethylbenzaldehyde (EBZD), and (**g**) the 4-ethylbenzaldehyde/benzaldehyde (EBZD/BZD) mixture (1:1). The visualization results of the (**b**) BZD, (**e**) EBZD, and (**h**) EBZD/BZD odor sources using heatmap images. The visualization results of the (**c**) BZD, (**f**) EBZD, and (**i**) EBZD/BZD odor sources processed using the Gaussian fitting model.

The visualization image of the BZD odor source placed at the central position was obtained by first decomposing the result matrix, $V_{1296\times 308}$, into a feature matrix, $H_{3\times 308}$, and a concentration matrix, $W_{1296\times 3}$. Three feature spectra in H were compared, as shown in Figure 3a. The spectrum of component r1 was identical to the SERS spectrum of BZD. The spectra of the other two components were considered to represent the sensor baseline and noise. In these two spectra, some obvious Raman peaks, including 900 cm^−1^, 1050 cm^−1^, and 1400 cm^−1^, were observed. In Figure 2a, obvious peaks can be observed at 900 cm^−1^, 1050 cm^−1^, and 1400 cm^−1^. We hypothesized that these characteristic peaks stemmed from the residual chemical agents present on the surface of the Ag NPs. This was due to our Ag NPs being synthesized via a chemical process without prior cleaning before their deposition onto the glass substrate. The values from the first column of the concentration matrix, W, which represented the coefficients for r1, were then normalized and reorganized into a 36 × 36 matrix, which was used to create a heatmap image (Figure 3b). In our sensor array, while there may not have been uniformity at the edges of individual SERS sensors, there was uniformity within each sensor’s surface. When detecting the same concentration of 4-ATP using nine SERS sensors, the average intensity values of 100 points collected on each substrate were very close, as shown in Figure 1f. This observation suggests the good uniformity of our sensors within the surface. The adsorption of gases evaporating from the BZD odor source onto the sensor involved a degree of randomness and could not perfectly represent the spatial distribution of the gas. In our detection experiment with minimal interference, the gas evaporated from the odor source was considered to follow a Gaussian distribution. Hence, the Gaussian fitting method was used to process the heatmap image and enhance the readability of the visualization results, enabling the localization of the odor source [39]. The standard deviation parameter of the Gaussian fitting model was adjusted to acquire a clearer visualization result. As shown in Figure 3c, a nearly circular shape with a higher concentration at the center and a lower concentration in the surroundings is obtained. Considering the open-top cylindrical container holding the odor solution, this visualization result corresponds with the ideal gas diffusion state.

In addition to the BZD odor source, the EBZD and EBZD/BZD mixed odor sources (1:1) were effectively visualized, as shown in Figure 3f,i. For the mixed odor source, the parameter r was set to 4 because there were two odors in the mixture. As a result, only one gas spectrum belonging to the EBZD/BZD mixture was extracted, as shown in Figure 3g. It could be explained that there were no separate spectra for the BZD and EBZD gas in the result matrix, V. For distinct odor sources, the component results after an NMF dimensionality reduction were distinct (Figure 3d,g). Additionally, the BZD and EBZD odor sources placed at the left-bottom corner were also visualized and localized, as shown in Figure S6. This confirmed that the NMF algorithm could extract the feature information (SERS spectra) of gases from the detection result matrix, V.

**Figure 4:**
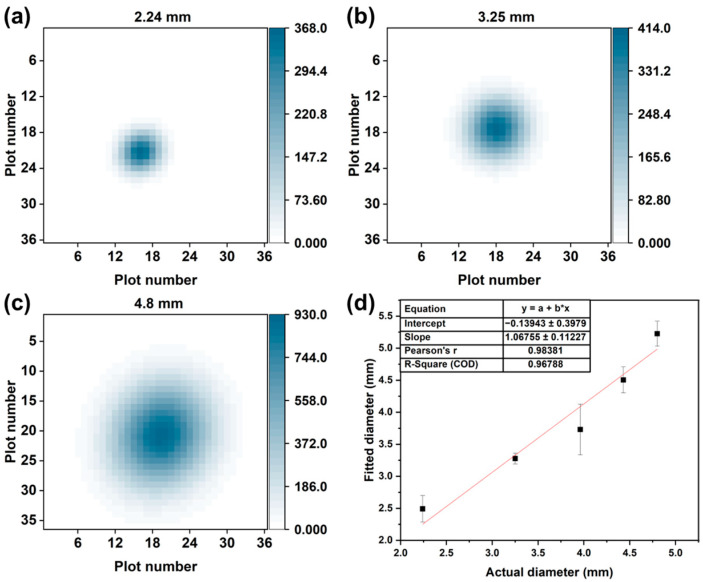
The visualization result of the benzaldehyde odor source using hole diameters of (**a**) 2.24, (**b**) 3.25, and (**c**) 4.8 mm. (**d**) The linear regression between the actual diameter and the fitted diameter.

The size of the odor source was estimated from the visualization result. The diameters of the holes on the aluminum plate were altered (2.24, 3.25, 3.96, 4.43, and 4.8 mm) to obtain BZD odor sources of different sizes. The spot size in the visualization result varied proportionately with the hole size on the plate, as illustrated in Figure 4a–c. In the Gaussian model, the parameters $\sigma_{x}$ and $\sigma_{y}$ varied with the gas spatial distribution. Therefore, these two values were utilized to estimate the spot size in the visualization result. The fitted diameter of the odor source was calculated using $(\sigma_{x}+$ $\sigma_{y})\;÷2$. Then, the fitted diameter was compared with the actual diameter of the hole, as depicted in Figure 4d. A linear regression was conducted to demonstrate the correlation between the fitted and actual diameters. An R^2^ value of 0.968 was obtained. Hence, the fitted diameter could be used to estimate the actual size of the odor source.

### 4.4. Visualization of Two BZD Odor Sources

**Figure 5:**
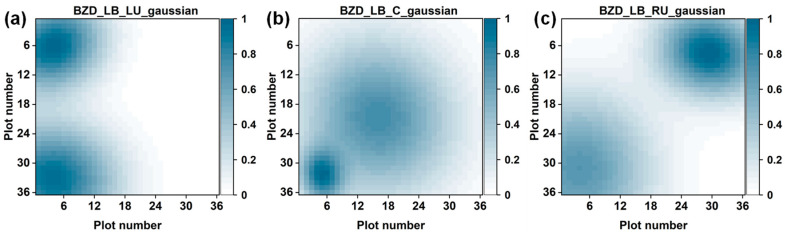
The visualization results of two benzaldehyde odor sources placed in three different position patterns: (**a**) left-bottom_left-up (LB_LU), (**b**) left-bottom_center (LB_C), and (**c**) left-bottom_right-up (LB_RU).

Two BZD odor sources were also simultaneously visualized using our proposed method. Three position patterns were defined for placing two BZD odor sources: left-bottom_ left-up (LB_LU), left-bottom_center (LB_C), and left-bottom_right-up (LB_RU). The visualization results of the three position patterns are shown in Figure 5. When the position patterns were LB_LU and LB_RU, the spatial distributions of the individual odor sources were nearly identical. Furthermore, there was little interference between the two odor sources because of the sufficient distance between them. Hence, the two odor sources could be distinctly discerned from the visualization results in each case. Regarding the LB_C position pattern, the area of the spatial distribution in the center was larger than that in the left-bottom corner. In the case of one odor source, we observed that the gas diffusion range of the odor source placed in the center was larger than that of the odor source placed in the bottom-left corner. Additionally, the distance between these two odor sources was closer compared with that in the other cases. Consequently, these two gas distributions were almost overlapping, but their positions were still localized.

### 4.5. Visualization and Identification of Two Distinct Odor Sources

**Figure 6:**
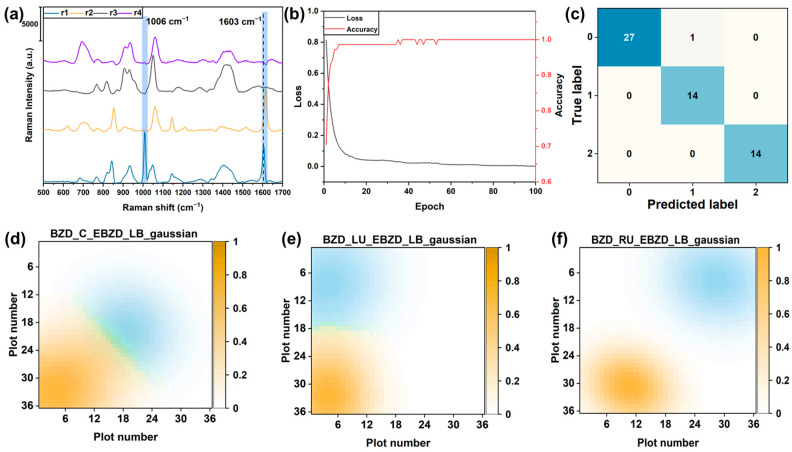
(**a**) Non-negative matrix factorization (NMF) components were obtained when benzaldehyde (BZD) and 4-ethylbenzaldehyde (EBZD) were detected using one sensor array. NMF components r1 and r2 strongly correspond to the BZD and EBZD SERS spectra, respectively. (**b**) The loss value of the training and valid datasets when the convolutional neural network (CNN) model was trained. (**c**) The confusion matrix result for the CNN model used for identifying the NMF components. (**d**–**f**) The visualization results of both BZD and EBZD odor sources detected by one SERS sensor array were obtained. The EBZD odor source was positioned in the left-bottom corner. The BZD odor source was positioned at the (**d**) center, (**e**) left-upper corner, and (**f**) right-upper corner.

The gas spatial distributions of the BZD and EBZD odor sources placed at different positions were visualized using one SERS sensor array. The visualization result was obtained using the processing method illustrated in Figure S7. The BZD and EBZD odor sources were placed in the center and the left-bottom positions. The SERS spectra from various spots were collected along the diagonal line of the sensor array, from the bottom-left to the top-right corners (Figure 2c). The SERS spectra of the BZD, EBZD, and EBZD/BZD gases were obtained. Thus, the detection result matrix, V, consisted of spectra information for the BZD and EBZD gases. Matrix, V, was decomposed using the NMF model with a parameter r of 4. The spectra of the BZD and EBZD gases were obtained in the feature matrix, as shown in Figure 6a.

To identify the component in the feature matrix result from the NMF model, a CNN recognition model was trained using the feature matrix obtained from the NMF model when one odor source was detected [46,47]. The trained CNN model was then used to identify the component in the feature matrix obtained when two distinct odor sources were detected. The spectra of the interference, BZD, and EBZD were labeled as 0, 1, and 2, respectively. As the training progressed, the loss gradually decreased, and the accuracy steadily improved, indicating that the model progressively enhanced its ability to fit the data during the learning process (Figure 6b). The effective identification of the confusion matrix result was obtained, as shown in Figure 6c, with a recognition accuracy of 98.21%. Hence, the spectra of these two gases were correctly identified using the trained CNN recognition model.

The concentration information of the gases was used to visualize the locations of the odor sources. First, separate images of the gas spatial distributions were obtained for these two gases, and then these two images were overlaid based on their corresponding positions to create a visualization image showing both gases simultaneously. Consequently, the identification and localization of two distinct odor sources were achieved, as depicted in Figure 6d. In this visualization result, the blue spot in the center represents the spatial distribution of the BZD gas, and the yellow spot represents the EBZD gas. Owing to the relatively close physical proximity of the two odor sources, an overlapping of the two spatial distributions was observed. In this overlapping section, we noted the emergence of spectra resulting from the mixture of the two gases (Figure 2c). Furthermore, the gas spatial distributions of these two odor sources placed in two other positions are shown in Figure 6e,f. There was no interaction between the spatial distributions when the distance between the two odor sources increased sufficiently.

This study’s limitations primarily involved two aspects: first, the presence of background peaks due to residual chemical agents on the sensor. These background peaks could hinder the detection of characteristic gas peaks in the SERS spectra. Second, there was a need to reduce the scanning time for the sensor array. In this study, spectral data were collected at a rate of one spectrum per point within one second, resulting in 1296 points collected across the array, thus requiring 1296 s for a complete array scan. However, for scenarios requiring swift visualizations, the time taken for a single visualization could be extensive, potentially not meeting the detection requirements.

## 5. Conclusions

In conclusion, a 2D SERS gas sensor array was constructed for identifying and visualizing the gas spatial distributions of BZD and EBZD odor sources. The SERS sensor with high reproducibility was fabricated using the self-assembly based on the Marangoni effect. The sensor array was placed above the odor source for gas adsorption. Then, the SERS spectra of the gases were obtained by scanning the surface of the sensor array. Differences in the spectra were observed at various positions. A detection result matrix was constructed using these spectra data and decomposed using the NMF model. The output result of the NMF model consisted of concentration and feature information for the detected gas. The gas spatial distribution was visualized using the concentration information plotted in a heatmap image. A Gaussian model was used to process this image for localizing the odor source at different positions. Furthermore, the size of the BZD odor source was correctly estimated using the final visualization result. A CNN recognition model was constructed to identify the different components in the feature information. As a result, the BZD and EBZD odor sources placed in different positions were simultaneously identified and localized using a single sensor array. This work provided a feasible method for visualizing the gas spatial distribution. Thus, it holds the potential application for identifying and exploring the location of odor sources in various fields.

In our future endeavors, we aim to address two primary challenges. Firstly, to develop SERS sensors with heightened sensitivity and devoid of distinct background Raman characteristic peaks. Such sensors would enable a quicker acquisition of SERS spectra at a single point, primarily showcasing the spectra related to the detected gases, facilitating rapid gas visualizations. Secondly, to increase the number of odor sources. While this experiment successfully visualized two odor sources simultaneously, real-world scenarios could involve multiple odors concurrently present in one location. Hence, identifying the constituents within more complex gas mixtures poses a greater challenge.

## Data Availability

Data are contained within the article and supplementary materials.

## Supplementary Materials

The following supporting information can be downloaded at: https://www.mdpi.com/article/10.3390/s24030790/s1, Figure S1: The fabrication process of the surface-enhanced Raman scattering (SERS) sensors, obtained by transferring the Ag nanoparticle (NP) monolayer film to the glass substrate; Figure S2: Five position patterns of the fixed odor sources; Figure S3: The process of gas evaporating from the odor source being adsorbed on the surface-enhanced Raman scattering (SERS) sensor array. The constructed sensor array (ii) was scanned using our program-controlled X-Y stage; Figure S4: The image of the program-controlled X-Y stage for the scanning system and SERS spectra collection system; Figure S5: The detection of the odor sources with different sizes. The size of the odor source was altered using an aluminum plate; Figure S6: The visualization results of the (a) benzaldehyde (BZD) and (c) 4-ethylbenzaldehyde (EBZD) odor sources using a heatmap image. The visualization results of the (b) BZD and (d) EBZD odor sources processed using the Gaussian fitting model. These two odor sources were positioned in the left-bottom corner; Figure S7: A flowchart of data processing to identify gases from the collected SRES spectra matrix and visualize the spatial distribution of the specific gas; Table S1: Vibrational mode assignments for 4-ATP.
