# Supplementary material for: Two-Dimensional SERS Sensor Array for Identifying and Visualizing the Gas Spatial Distributions of Two Distinct Odor Sources
Source: Sensors (Basel). 2024 Jan 25;24(3):790. doi: 10.3390/s24030790 (PMC10857130; doi:10.3390/s24030790)
Supplement: Supplementary file 1 [file sensors-24-00790-s001.zip › sensors-2762695-supplementary.pdf]

Two-Dimensional SERS Sensor Array for Identifying and Visualizing the Gas Spatial Distributions of Two Distinct Odor Sources

Lin Chen [1,†], Hao Guo [2,†], Cong Wang [2], Bin Chen [3], Fumihiro Sassa [2] and Kenshi Hayashi [2,*]

1 Department of Information Science, Joint Graduate School of Mathematics for Innovation, Kyushu University, Fukuoka 819-0395, Japan
2 Department of Electronics, Graduate School of Information Science and Electrical Engineering, Kyushu University, Fukuoka 819-0395, Japan; guo.hao.097@s.kyushu-u.ac.jp (H.G.); cong.929@s.kyushu-u.ac.jp (C.W.); sassa@ed.kyushu-u.ac.jp (F.S.)
3 Chongqing Key Laboratory of Non-Linear Circuit and Intelligent Information Processing, College of Electronic and Information Engineering, Southwest University, Chongqing 400715, China; chenbin121@swu.edu.cn
* Correspondence: hayashi@ed.kyushu-u.ac.jp

† These authors contributed equally to this work.

Experimental method

1. Instrumentation

The SERS sensor was fabricated using an SVC-700TMSG/Adexcel Ag sputtering machine (Sanyu Electron Co., Ltd., Japan). The morphology of the sensor was measured using an SU8000 scanning electron microscope (Hitachi High-Tech, Japan). UV-vis transmission spectra were measured using a UV1800 spectrophotometer (Shimadzu, Japan). A custom gas sensing cell was fabricated using an UltimakerS5 3D printer (Ultimaker, Netherlands). Raman spectra were acquired using an AvaRaman portable Raman spectrometer (Avantes, Netherlands) equipped with a 532 nm excitation laser light source (power 1.5 mW). The data were analyzed using Python version 3.6 software.

2. Synthesis of the Ag NP seed solution

First, the Ag nanoparticle (NP) seed solution was synthesized. Herein, 1 mL of the aqueous sodium citrate solution (1 wt%), 0.25 mL of the aqueous silver nitrate ($AgNO_3$) solution (1 wt%), and 0.2 mL of the aqueous sodium chloride (NaCl) solution (20 mM) were added to 1.05 mL of water whilst stirring. After 5 min, the citrate–silver–NaCl

solution was added to 47.5 mL of boiling water, but 80 uL of the aqueous L-ascorbic acid (AA) solution was added into the boiling water 1 min before the citrate–silver–NaCl solution. After heating for 1 h, the Ag NP seed solution was finally obtained.

3. Synthesis of Ag NPs with a size of 90 nm

First, 2 mL of the aqueous $AgNO_3$ solution (1 wt%) was mixed with 800 μL of ammonia water (25%–28%). Next, 550 μL of the original solution of Ag NP seeds was added into the water (18.92 mL) whilst stirring in a 50 mL glass bottle. Then, the aqueous silver–ammonia complex solution (280 μL, 43 mM) and aqueous AA solution (8 mL, 2.5 mM) were added into a 50 mL glass bottle. After stirring for 1 h, the Ag NPs were concentrated by centrifugation and redispersed in 5 mL of the aqueous solution. Finally, 100 uL of the aqueous tetrabutylammonium nitrate ($C_{16}H_{36}N_2O_3$) solution was added.

4. Fabrication of SERS sensors using the Ag NP solution

First, 2.5 mL of the concentrated aqueous Ag NP solution was mixed with 2 mL of chloroform in a 15 mL centrifuge tube. Then, the tube was shaken for 30 s by hand. After that, 0.6 uL of hexane was added to the tube. The formation of a densely packed NP monolayer film occurred at the interface between hexane and water in this solution. Finally, the monolayer film was transferred to the glass substrate as the SERS sensor.

5. Construction of the Gaussian fitting model

The shape of the gas evaporating from the odor source on the SERS sensor array was almost circular or elliptical. Therefore, the spatial distribution was fitted using a 2D Gaussian function, which can be expressed as [39]:

$$I(x,y) = A\exp(-(a(x-x_0)^2 + 2b(x-x_0)(y-y_0) + c(y-y_0)^2))$$

$$a = \frac{cos^2\theta}{2{\sigma_x}^2} + \frac{sin^2\theta}{2{\sigma_y}^2}$$

$$b = -\frac{\sin 2\theta}{4{\sigma_x}^2} + \frac{\sin 2\theta}{4{\sigma_y}^2}$$

$$c = \frac{sin^2\theta}{2{\sigma_x}^2} + \frac{cos^2\theta}{2{\sigma_y}^2}$$

Here, (x,y) is the coordinate, A is the amplitude, x_0 and y_0 are the coordinates of the center point, σ_x and σ_y are the standard derivation components, and θ is the rotation angle. These parameters were fitted to construct a Gaussian distribution, and the result was used to create a visualization image of the gas spatial distribution.

Supplementary figures and tables

Figures

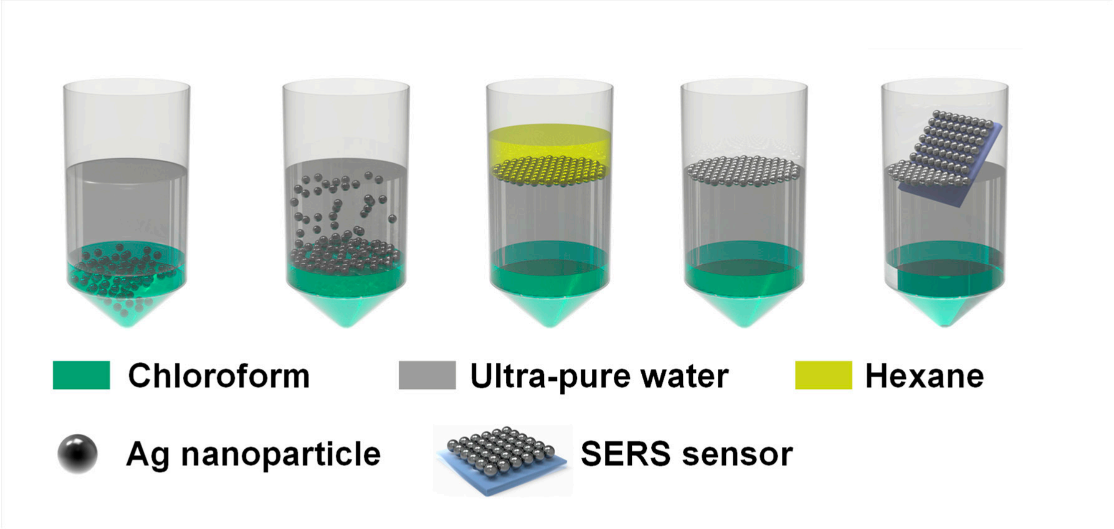

Figure S1. The fabrication process of the surface-enhanced Raman scattering (SERS) sensors, obtained by transferring the Ag nanoparticle (NP) monolayer film to the glass substrate.

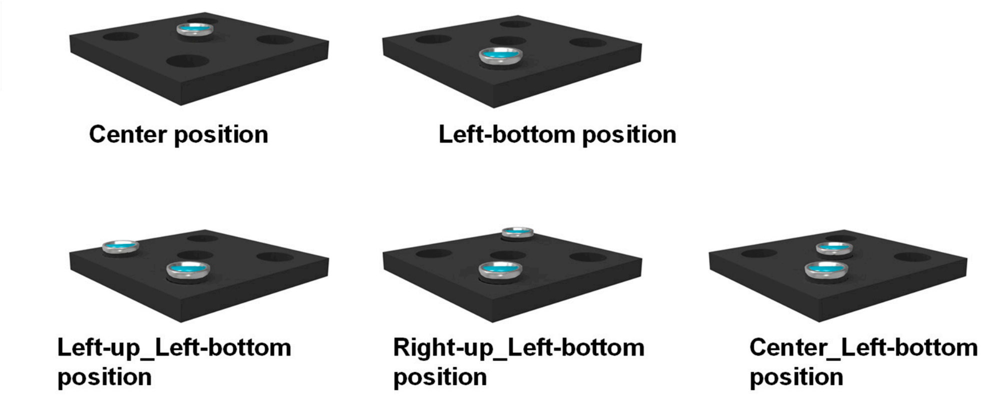

Figure S2. Five position patterns of the fixed odor sources.

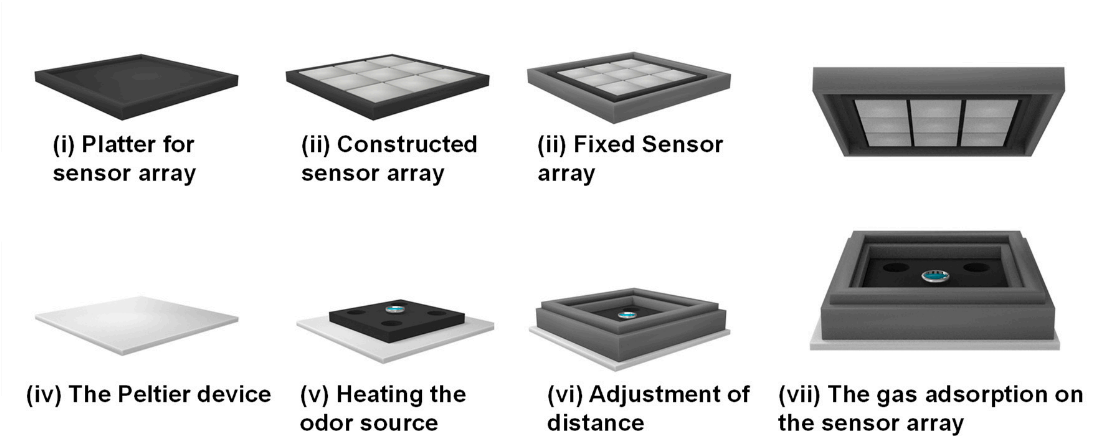

Figure S3. The process of gas evaporating from the odor source being adsorbed on the surface-enhanced Raman scattering (SERS) sensor array. The constructed sensor array (ii) was scanned using our program-controlled X-Y stage.

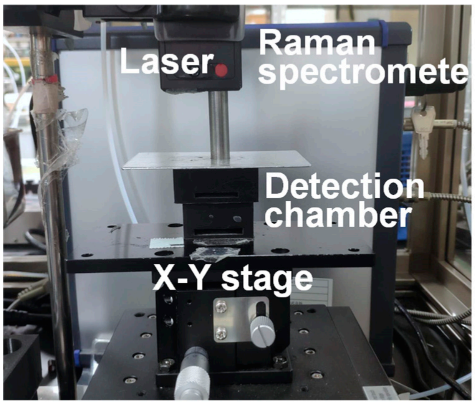

Figure S4. The image of the program-controlled X-Y stage for the scanning system and SERS spectra collection system.

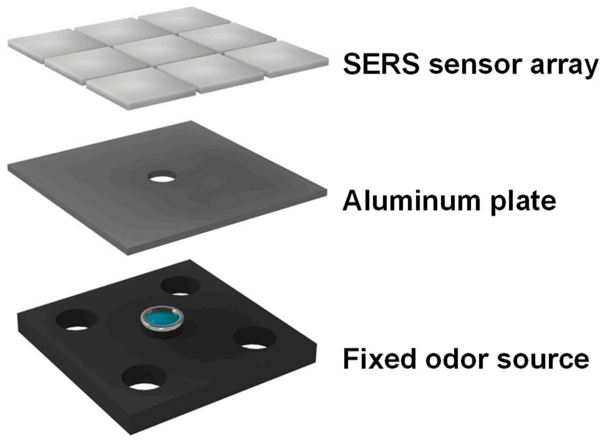

Figure S5. The detection of the odor source with different sizes. The size of the odor source was altered using an aluminum plate.

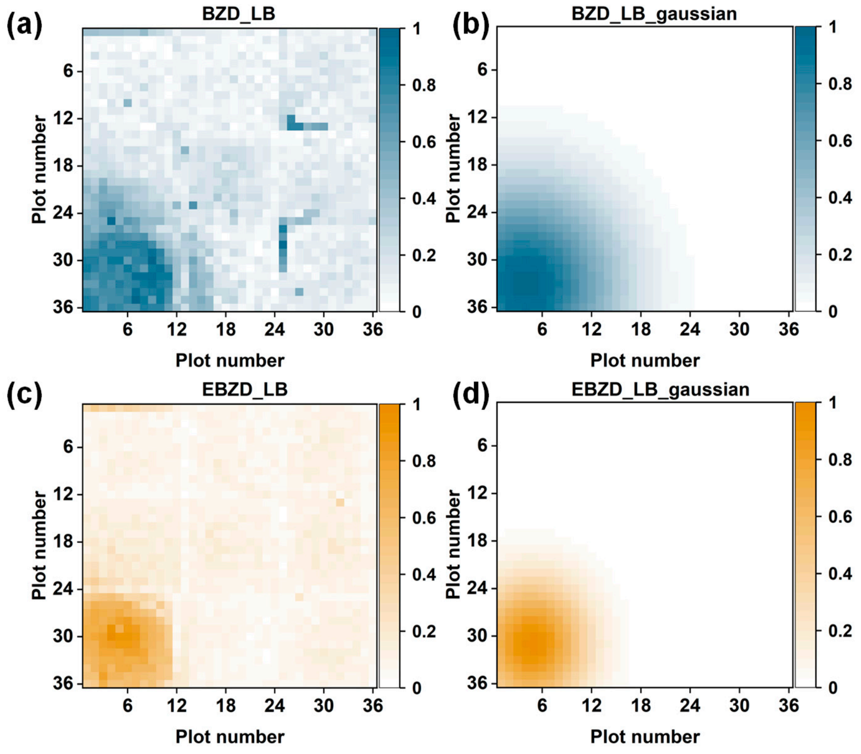

Figure S6. The visualization results of the (a) benzaldehyde (BZD) and (c) 4-ethylbenzaldehyde (EBZD) odor sources using a heatmap image. The visualization results of the (b) BZD and (d) EBZD odor sources processed using the Gaussian fitting model. These two odor sources were positioned at the left-bottom corners.

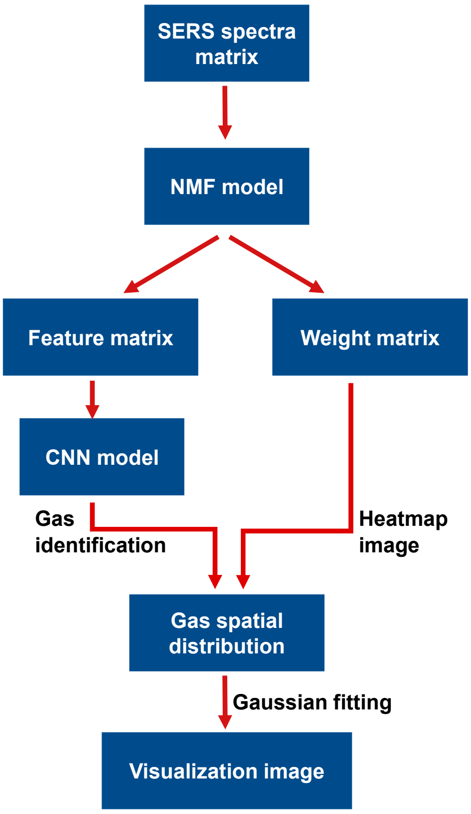

Figure S7. A flowchart of data processing to identify gases from the collected SRES spectra matrix and visualize the spatial distributions of the specific gas.

Tables

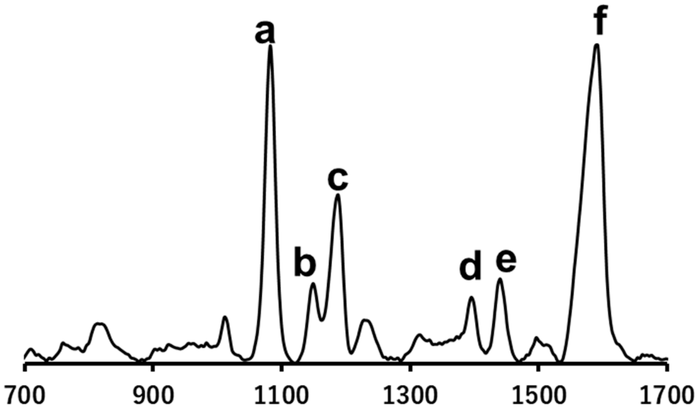

a	1082	v(C=C) + v(C-S)
b	1148	β(C-H) + v(C-N)
c	1181	β(C-H)
d	1394	v(N=N) + v(C-N)
e	1440	v(N=N) + β(C-H)
f	1585	v(C=C)

Table S1. Vibrational mode assignments for 4-ATP.

Reference

39. Shi, J.C.; Yang, Z.F.; Wu, L.X.; Niu, J.J. Large-Gradient Interferometric Phase Unwrapping Over Coal Mining Areas Assisted by a 2-D Elliptical Gaussian Function. IEEE Geoscience and Remote Sensing Letters 2022, 19, 1-5, doi:10.1109/LGRS.2022.3223627.
